# The Neurogenic Abnormities of Paraspinal Muscles Lead to Asymmetry of Fibre Types in Adolescent Idiopathic Scoliosis

**DOI:** 10.1111/jcmm.70619

**Published:** 2025-05-24

**Authors:** Tianyuan Zhang, Bin Li, Wenyuan Sui, Xiexiang Shao, Yaolong Deng, Zifang Zhang, Jingfan Yang, Zifang Huang, Sheng Li, Xin Fu, Wenjun Yang, Junlin Yang, Ping Hu

**Affiliations:** ^1^ Spine Center Xinhua Hospital Affiliated to Shanghai Jiaotong University School of Medicine Shanghai China; ^2^ Department of Spine Surgery The Third Affiliated Hospital of Sun Yat‐Sen University Guangzhou Guangdong China; ^3^ Xin Hua Hospital Affiliated to Shanghai Jiao Tong University School of Medicine Shanghai China; ^4^ The 10th People's Hospital Affiliated to Tongji University Shanghai China; ^5^ Guangzhou Laboratory‐Guangzhou Medical University Guangdong China; ^6^ Key Laboratory of Biological Targeting Diagnosis, Therapy and Rehabilitation of Guangdong Higher Education Institutes The Fifth Affiliated Hospital of Guangzhou Medical University Guangdong China

**Keywords:** adolescent idiopathic scoliosis, neurogenic abnormities, neuromuscular junctions, paraspinal muscles

## Abstract

The aetiology of adolescent idiopathic scoliosis (AIS) is not clear, and may involve disorders in multiple systems. This study aims to perform the morphological and molecular analysis of neuromuscular junctions (NMJs) and explore the asymmetry of paraspinal muscles in AIS. We collected paraspinal muscles from AIS patients during surgery and also enrolled congenital scoliosis (CS) and non‐scoliosis patients as controls. We performed immunofluorescence staining of NMJs for morphological analysis. Then, we extracted NMJs regions for further validation at the molecular level. We also explored the neurogenic abnormalities in the convex side and compared the asymmetry of paraspinal muscles. Morphological analysis of NMJs showed that the nerve terminal‐related variables in the convex side were significantly decreased. The expression of denervation markers was increased in the synapse‐rich regions. The expression of denervation markers in the convex paraspinal muscles was also significantly increased. Compared with CS and non‐scoliosis patients, paraspinal muscles of AIS exhibited the transformation of fibre types, characterised by an increase in the proportion of type I fibres in the convex side. The phenomenon of fibre‐type grouping was also noted, confirming the presence of neurogenic abnormalities. This study first investigated the morphological and molecular disorders of NMJs in the paraspinal muscles from AIS patients. We found that the neurogenic abnormalities existed in the convex side of the paraspinal muscle, which could lead to the conversion and grouping of fibre types. This resulted in an imbalance of bilateral paraspinal muscles and might be a potential driver of scoliosis.

## 1 Introduction

Adolescent idiopathic scoliosis (AIS) is the most common form of structural spinal deformity and affects around 1%–4% of adolescents all over the world [1]. Unlike congenital scoliosis (CS), degenerative scoliosis and other types of scoliosis with clear pathogenic factors, AIS develops curves in otherwise healthy individuals [2, 3]. Although many hypotheses have been proposed, including genetics, the nervous system, paraspinal muscles, biomechanics, skeletal spinal growth and environmental factors, the exact aetiology remains unknown [4, 5, 6]. Among them, the asymmetry and imbalance of paraspinal muscles play a vital role in spinal stability and alignment [7]. However, some research argues that it is secondary to the onset of scoliosis [8]. Recently, with advances in magnetic resonance imaging (MRI) technology, considerable neuromorphological abnormalities have been found in the brain and spinal cord of AIS patients [9, 10, 11]. For example, Kong et al. found variation in anisotropy and diffusivity along the medulla oblongata and the whole spinal cord in AIS using diffusion tensor imaging [12]. Domenech et al. revealed abnormal activation of the motor cortical network in AIS by functional MRI [13]. In addition, other studies have demonstrated postural instability, abnormal proprioceptive function and abnormal somatosensory‐evoked potentials in AIS [14, 15, 16]. These pieces of evidence support that the neuromuscular factors might be closely involved in the pathogenesis of AIS, but the exact mechanism still remains to be elucidated.

The neuromuscular junction (NMJ) is a peripheral synapse that conveys neural signals from lower motor neurons to skeletal muscle fibres [17]. It plays an essential role in controlling muscle contractions. Improper NMJ formation and maintenance have been implicated in various neuromuscular disorders, such as Duchenne muscular dystrophy (DMD) and spinal muscular atrophy (SMA) [18, 19, 20]. Therefore, monitoring the morphology of NMJs and their alterations represents a valuable tool for pathogenetic studies [21]. Interestingly, these NMJ diseases often contribute to the onset of scoliosis. For example, Kinali et al. found that about 75%–90% of patients with DMD would develop scoliosis [22]. Wijngaarde et al. observed that the lifetime probability of receiving scoliosis surgery was almost 80% in SMA type 1c and 2 [23]. Recently, Wang et al. concluded that impaired neurotransmission caused by a mutation of the SLC6A9 gene may contribute to AIS and that scoliosis was a subtle phenotype of neuromuscular coordination deficits [24]. This provided further clues that neuromuscular disorders played a crucial role in the aetiology of AIS. However, no previous studies had thoroughly examined the morphological changes of NMJs in AIS patients. To solve this problem, we followed a standardised workflow to perform the quantitative morphological analysis of NMJs from bilateral paraspinal muscles and investigated their potential role in AIS. We also collected samples from CS and non‐scoliosis patients to make a comparison.

## 2 Results

### 2.1 Abnormal Morphological Variables of NMJs in AIS

**FIGURE 1:**
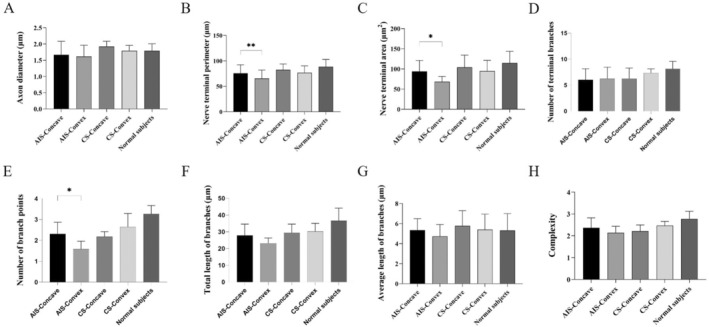
Comparison of pre‐synaptic morphological variables. (A) Axon diameter; (B) nerve terminal perimeter; (C) nerve terminal area; (D) number of terminal branches; (E) number of branch points; (F) total length of branches; (G) average length of branches; (H) complexity. *p < 0.05, **p < 0.01.

**FIGURE 2:**
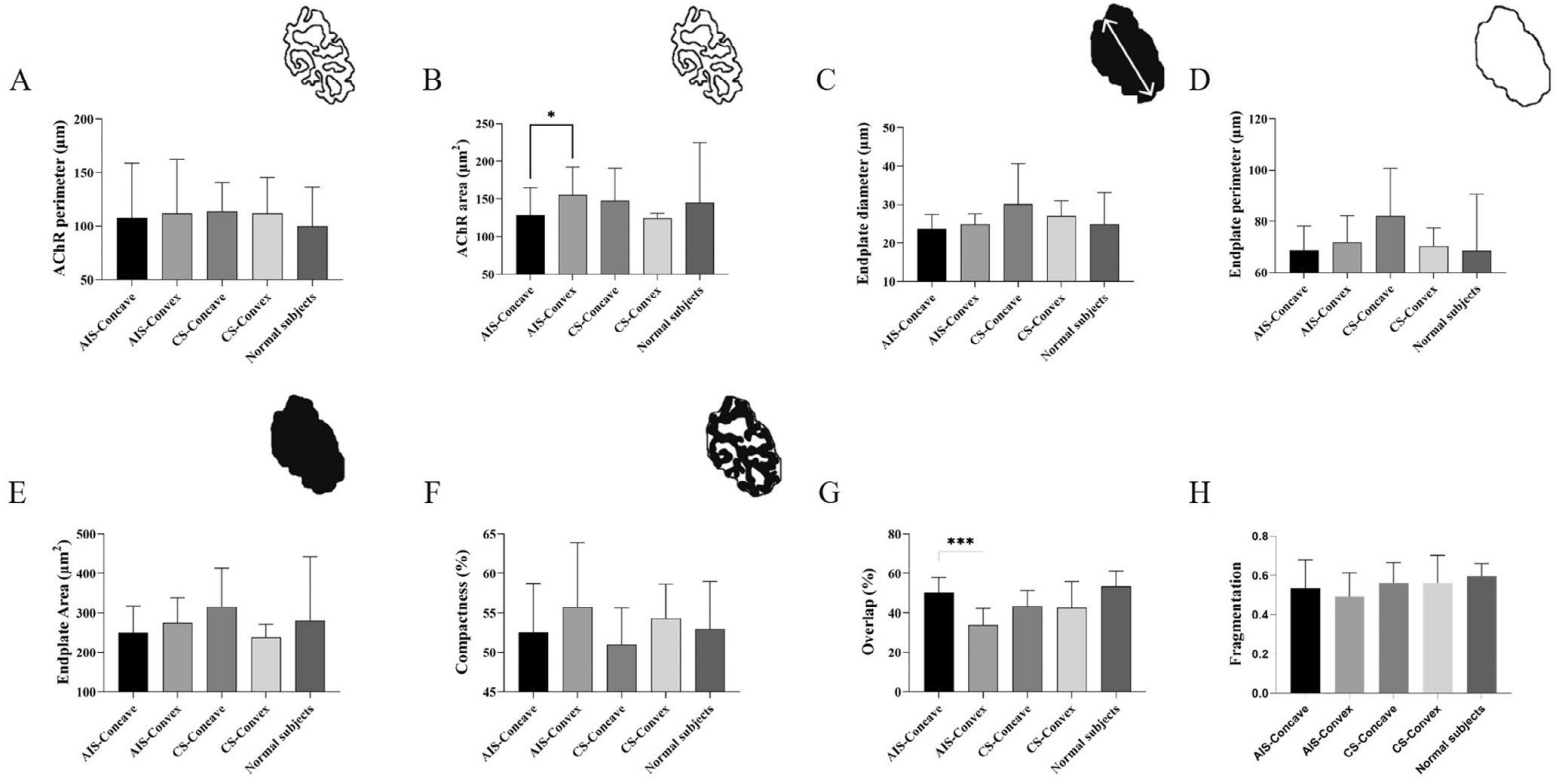
Comparison of post‐synaptic and derived morphological variables. (A) AChR perimeter; (B) AChR area; (C) endplate diameter; (D) endplate perimeter; (E) endplate area; (F) compactness; (G) overlap; (H) fragmentation. *p < 0.05, ***p < 0.001.

**FIGURE 3:**
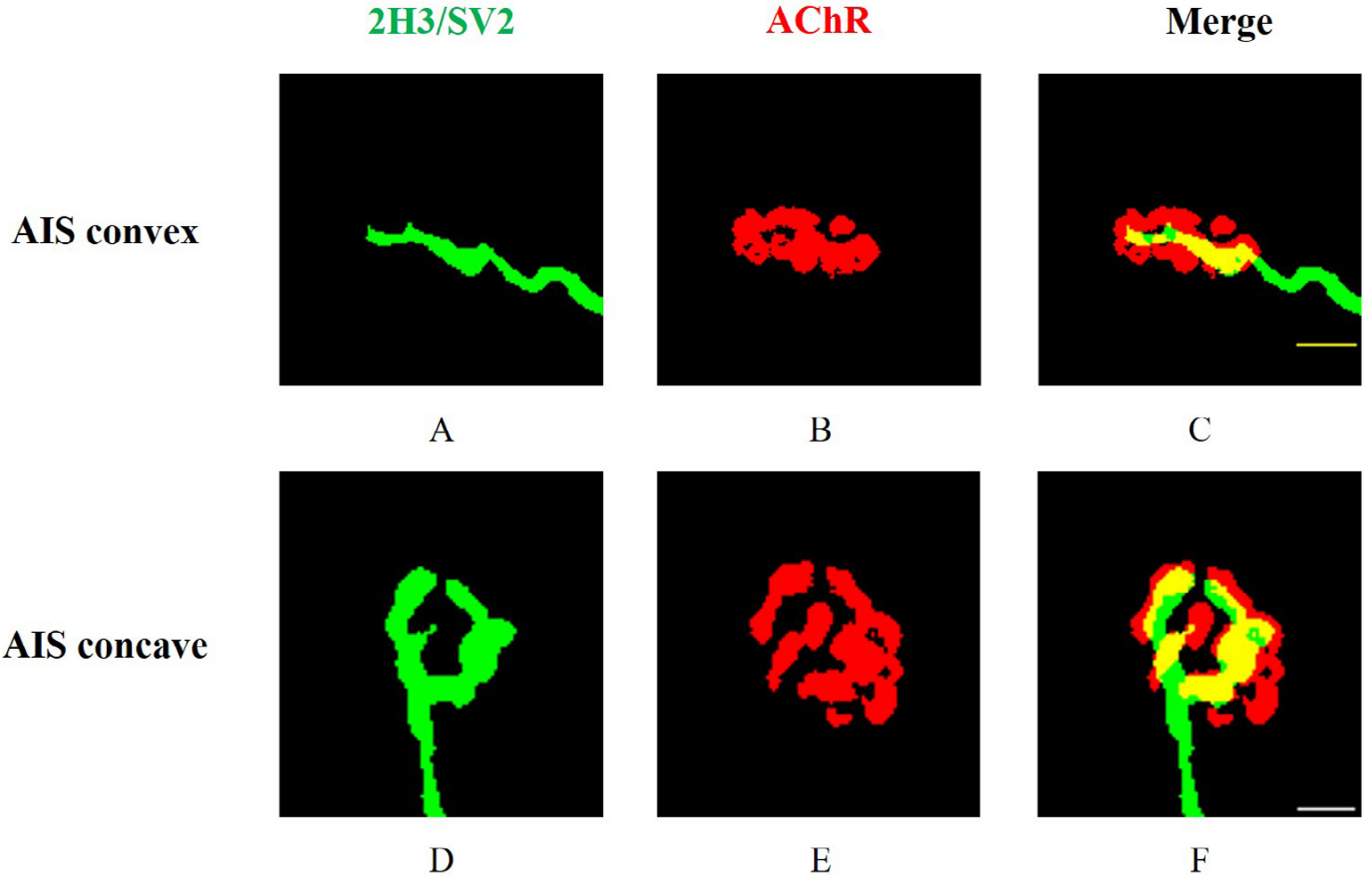
The representative picture of immunofluorescent staining of NMJs in AIS. (A–C) NMJ in the convex side of paraspinal muscles; (D–F) NMJ in the concave side of paraspinal muscles. 2H3/SV2 = neurofilament/synaptic vesicles, green fluorescence stains pre‐synapse, red fluorescence stains post‐synapse, bar equals 10 μm.

In the morphological analysis of NMJs, we enrolled 10 AIS, 5 CS and 5 non‐scoliosis patients. CS patients were used to investigate the potential changes of NMJs secondary to scoliosis, and non‐scoliosis patients were used as normal controls. The baseline information about them is shown in Table 1. There was no significant difference among the three groups. Qualitatively, rough observations of all three groups revealed that NMJs from paraspinal muscles have the normal ‘nummular’ morphological appearance. Then, we followed the ‘NMJ‐morph’ workflow to carry out the comprehensive morphological analysis of NMJs. The overview of NMJs' morphological variables was presented in Table S2. In AIS, nerve terminal perimeter, nerve terminal area, number of branch points and overlap in the convex side of paraspinal muscles were significantly decreased compared to those in the concave side (Figure 1). For post‐synaptic variables, AChR area and average area of AChR clusters were significantly increased in the convex side. However, these variables showed no difference in the bilateral paraspinal muscles of CS (Figure 2). Compared to normal controls, one‐way ANOVA demonstrated that nerve terminal area, number of branch points, total length of branches, complexity and overlap were significantly different among the three groups. This result implied the existence of primary neurogenic abnormalities of NMJs in the convex side of paraspinal muscles for AIS patients (Figure 3A–F).

**TABLE 1:** The baseline clinical information of this study.

	AIS	CS	Normal controls	*p*
Age (years)	13.38 ± 1.69	14.00 ± 1.58	14.75 ± 1.71	0.417
Sex				
Male	2	1	2	0.807
Female	8	4	3	
Height (cm)	155.63 ± 6.95	157.80 ± 8.17	157.50 ± 6.45	0.844
Weight (kg)	41.53 ± 5.23	46.40 ± 6.11	52.25 ± 8.58	0.045
BMI (kg/m^2^)	17.12 ± 1.52	18.66 ± 2.19	20.94 ± 2.15	0.016
Main curve (°)	62.50 ± 11.89	65.00 ± 5.00	—	0.668

Abbreviations: AIS = adolescent idiopathic scoliosis, BMI = body mass index, CS = congenital scoliosis.

### 2.2 Elevated Expression of Denervation Markers in Synapse‐Rich Regions

**FIGURE 4:**
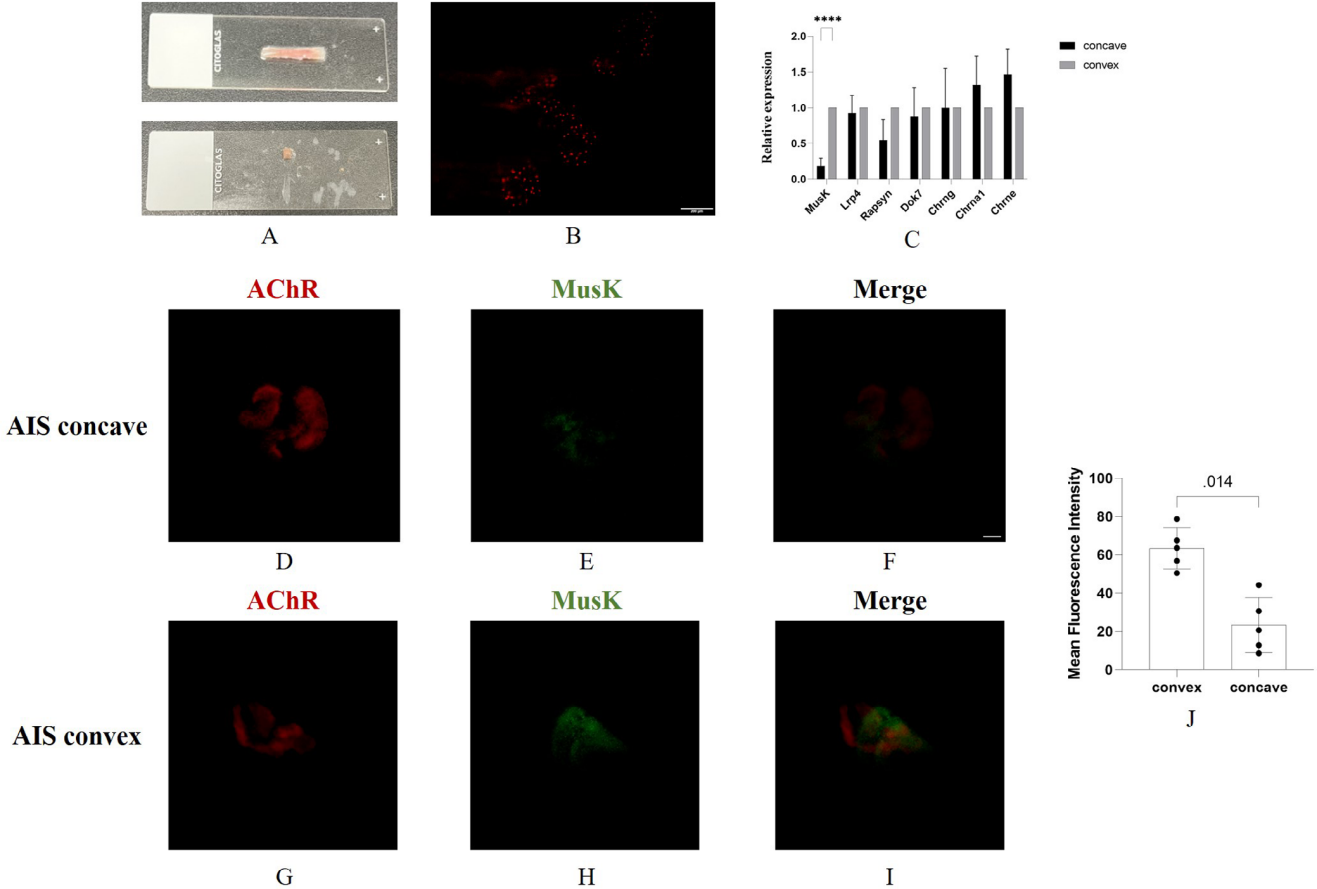
Expression of target genes in the synapse‐rich regions. (A) Dissecting the synapse‐rich region from the long‐stripe blocks of paraspinal muscles; (B) immunofluorescent staining of the synapse‐rich region; (C) comparison of target genes in the synapse‐rich regions between both sides of paraspinal muscles; (D–F) representative picture of immunofluorescent staining of MuSK in the concave side; (G–I) representative picture of immunofluorescent staining of MuSK in the convex side; (J) comparison of mean fluorescence intensity of MuSK between both sides. Red fluorescence stains AChRs, green fluorescence stains MuSK, bar equals 10 μm, ****p < 0.0001.

To explore the changes of NMJs from the molecular level, we next extracted the synapse‐rich regions from the long‐stripe blocks of paraspinal muscles (Figure 4A). The immunofluorescent staining of AChRs by α‐bungarotoxin confirmed the locations of NMJs (Figure 4B). The expression of key genes that formed the structure of NMJs, including MuSK, Lrp4, Rapsyn, Dok7, Chrng, Chrna1 and Chrne, was examined by qPCR on the dissected synapse‐rich regions. Chrng, Chrna1 and Chrne were different subunits of AChRs, and Chrng encoded the gamma subunit in fetal and denervated muscle. MuSK, Chrng, Chrna1 and Chrne were distributed at the post‐synapse regions of NMJs, and a marked up‐regulation of these denervation‐responsive transcripts indicated pre‐synaptic denervation [25]. The results of qPCR showed that the expression of MuSK in the convex side was significantly increased compared to the concave side, while other genes showed no significant difference (Figure 4C). The immunofluorescent staining of MuSK was further performed to validate its expression. The result showed that the mean fluorescence intensity of MuSK in the convex side was also significantly elevated compared to the concave side (Figure 4D–J). This finding suggested that there was no major molecular mutation in the NMJs and that the elevated expression of MuSK was a compensatory response to the neurogenic abnormalities in the synapse‐rich regions.

### 2.3 Existence of Neurogenic Abnormalities in the Convex Side of Paraspinal Muscles

**FIGURE 5:**
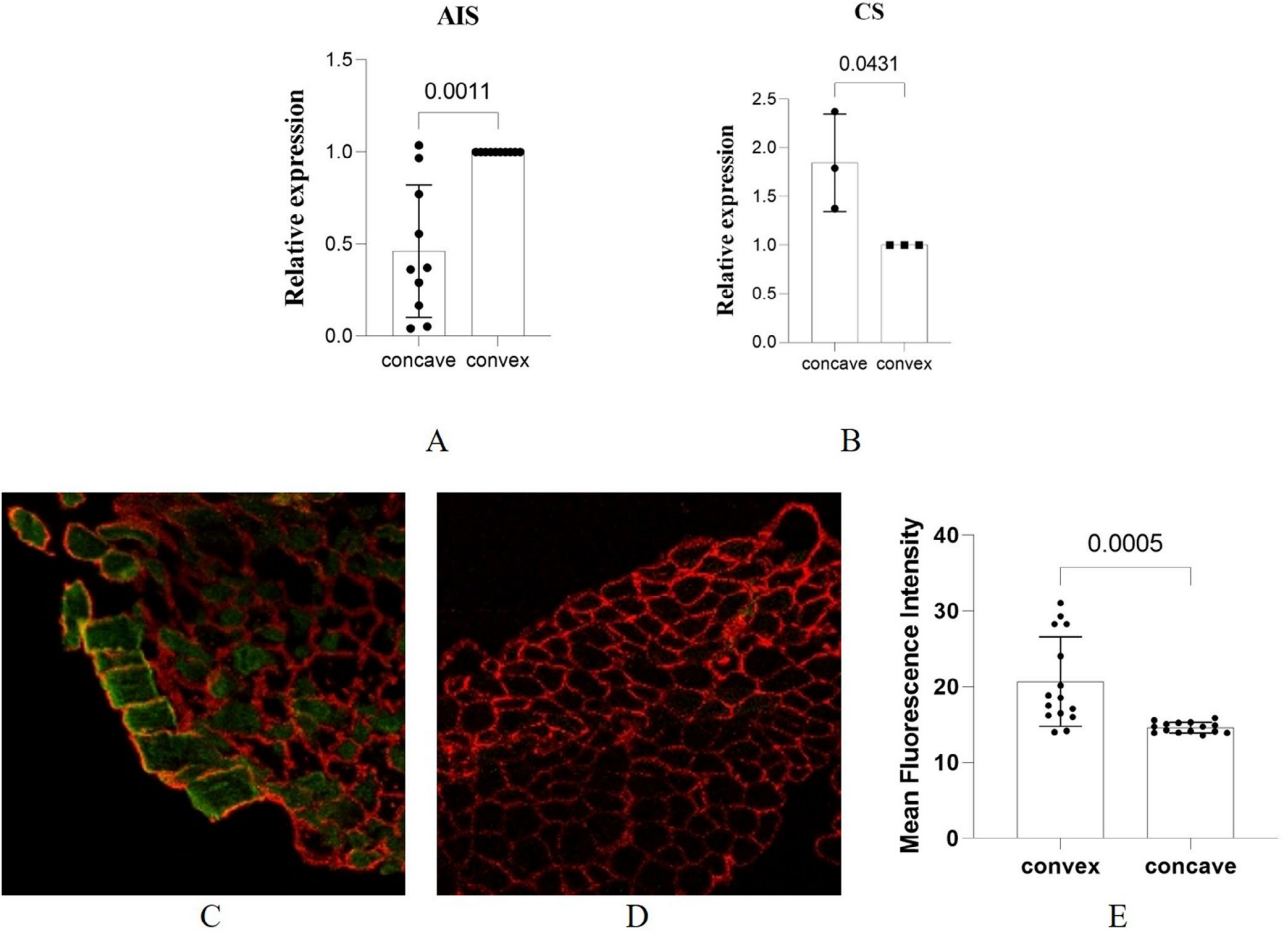
Expression of denervation markers between both sides of paraspinal muscles. (A) mRNA expression of NCAM in paraspinal muscles of AIS; (B) mRNA expression of NCAM in paraspinal muscles of CS; (C) immunofluorescent staining of NCAM in the convex paraspinal muscles of AIS; (D) immunofluorescent staining of NCAM in the concave paraspinal muscles of AIS; (E) comparison of mean fluorescence intensity of NCAM between both sides of paraspinal muscles in AIS. Green fluorescence stains NCAM‐positive fibres, red fluorescence stains laminin.

To validate the neurogenic abnormalities in the paraspinal muscles of AIS, we collected muscle samples from AIS and CS patients to detect the expression of neural cell adhesion molecule (NCAM), which was the most commonly used biomarker of muscle denervation. The results of qPCR showed that, for AIS patients, the expression of NCAM in the convex side of paraspinal muscles was significantly higher than in the concave side (Figure 5A, *p* = 0.0011). However, for CS patients, the expression of NCAM in the concave side was significantly elevated (Figure 5B, *p* = 0.0431), which indicated a secondary outcome of scoliosis. The different results demonstrated that the neurogenic abnormality in the convex side of the paraspinal muscle may be the primary driver of scoliosis for AIS patients. Next, we collected another five muscle samples of AIS patients to perform the quantitative immunofluorescent staining and study the distribution of NCAM. The results demonstrated that the signal of NCAM was positive in the convex side, while it was barely shown in the concave side (Figure 5C,D). Three random fields per sample were selected to measure. The mean fluorescence intensity of NCAM in the convex side was significantly higher than the concave side (Figure 5E, *p* = 0.0005). These results further confirmed the existence of neurogenic abnormalities in the convex side of paraspinal muscles for AIS patients.

### 2.4 Fibre Type Shift in the Convex Side of Paraspinal Muscles

**FIGURE 6:**
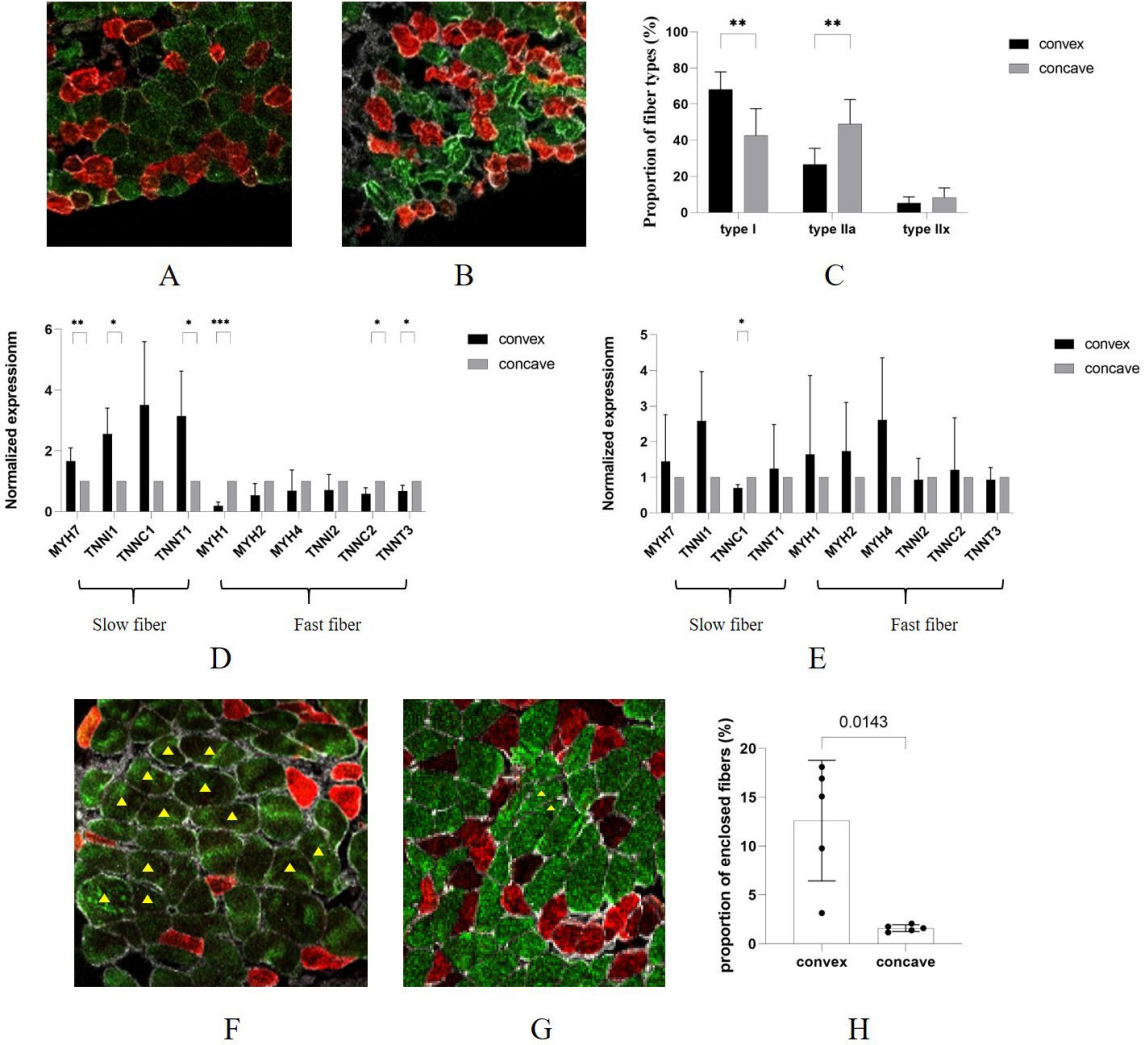
Analysis of proportion and distribution of fibre types. (A) Immunofluorescent staining of fibre types in the convex paraspinal muscles of AIS; (B) immunofluorescent staining of fibre types in the concave paraspinal muscles of AIS; (C) comparison of proportion of fibre types between both sides of paraspinal muscles in AIS; (D) comparison of mRNA expression of slow and fast fibre genes between both sides of paraspinal muscles in AIS; (E) comparison of mRNA expression of slow and fast fibre genes between both sides of paraspinal muscles in CS; (F) analysis of fibre‐type grouping in the convex paraspinal muscles of AIS, yellow triangles indicates enclosed fibres; (G) analysis of fibre‐type grouping in the concave paraspinal muscles of AIS, yellow triangles indicates enclosed fibres; (H) comparison of proportion of enclosed fibres between both sides of paraspinal muscles in AIS. Green fluorescence stains type I fibre, red fluorescence stains type IIa fibre, white fluorescence stains laminin, *p < 0.05, **p < 0.01, ***p < 0.001.

The innervation status of skeletal muscles determines their specific fibre type. To study the potential impacts of neurogenic abnormalities on fibre types, we first collected three samples of non‐scoliosis controls to investigate the normal distribution of fibre types (Table S3). For the control group, we found that type I fibre was dominant in the paraspinal muscles, and there was no difference in distribution between the two sides (Figure S1). Then, we collected five AIS samples and measured 955.8 ± 512.0 fibres per sample (Table 2). The results showed that the proportion of type I fibre was significantly increased in the convex side, while the proportion of type IIa fibre was significantly increased in the concave side (Figure 6A–C). Furthermore, we tested the expression of myofibre‐related genes in AIS and CS. The results proved the asymmetry of fibre distribution in AIS, but no such asymmetry in CS (Figure 6D,E). Finally, it was worth mentioning that the phenomenon of fibre‐type grouping was found in the convex side of paraspinal muscles, which was also an indication of neurogenic muscle disorders (Figure 6F,G). We measured the prevalence of the enclosed fibre to evaluate the extent of fibre type grouping in five AIS patients. The results showed that the prevalence of the enclosed fibre was 12.61% ± 5.52% in the convex side, but significantly decreased to 1.61% ± 0.31% in the concave side (Figure 6H). It demonstrated the prevalent occurrence of fibre‐type grouping, further providing evidence of neurogenic abnormalities in the convex side of paraspinal muscles.

**TABLE 2:** Proportion of different fibre types between both sides of the paraspinal muscles in AIS.

	Concave	Convex	*p*
Proportion of type I fibre (%)	42.7 ± 12.9	68.1 ± 8.5	0.004
Proportion of type IIa fibre (%)	49.0 ± 11.7	26.7 ± 7.7	0.005
Proportion of type IIx fibre (%)	8.3 ± 4.6	5.2 ± 2.9	0.082

## 3 Discussion

Despite decades of research, the primary cause of AIS still remains unknown, and many arguments exist. Recently, the theory of neuromuscular dysfunction, such as the imbalance of paraspinal muscles and neurological deficits, has attracted increasing attention. However, the relationship between them and how they drive scoliosis remains perplexing. According to the literature, NMJs are critical to controlling muscle function, and disorders of NMJs could destroy the intimate interaction between motor nerve terminals and muscle fibres. Therefore, abnormal NMJs of the paravertebral muscles would easily influence spinal stability and balance. For example, for patients with scoliosis associated with syringomyelia, Zhu et al. found abnormal diffusion of AChRs at extra‐junctional sites and the existence of the immature gamma‐AChR subunit in the paravertebral muscle [26]. Besides, Theroux et al. reported that there was an aberrant distribution of AChRs relative to the acetylcholinesterase found at the NMJs of paravertebral muscles in patients with cerebral palsy and concomitant scoliosis [27]. However, no studies have thoroughly explored whether there were disorders of NMJs in the paravertebral muscles of AIS, even if many researchers provided evidence of neuromuscular pathogenic factors. Herein, we hypothesised that the NMJs of paravertebral muscles in AIS might be affected to some extent and carried out this study to compare the morphology of NMJs between the two sides of paravertebral muscles in AIS patients.

In this study, we first observed the aberrant nerve terminals of NMJs in the convex side of paraspinal muscles, and further provided sufficient evidence of neurogenic abnormalities by increased expression of biomarkers and the phenomenon of fibre‐type grouping. We also excluded the potential alterations secondary to the onset of scoliosis in CS patients. The primary neurogenic abnormalities in the convex side of paraspinal muscles indicated impaired capability of nerve conduction and muscle contraction, thereby driving the formation of scoliosis. Consistent with our hypothesis, Liu et al. evaluated the biomechanical features of paravertebral muscles in AIS and found that muscle tone and stiffness on the convex side were significantly weaker than those on the concave side. They also concluded that the asymmetric biomechanical characteristics of paravertebral muscles are closely related to the severity of scoliosis. The myoelectric activity measured by surface electromyogram (EMG) could also reflect the neuromuscular function of paraspinal muscles [28]. However, previous studies that focused on this did not reach a consensus. A recent review screening 94 related studies summarised that for EMG amplitude, 43 outcomes provided evidence of convex > concave activation, 85 outcomes supported no difference between sides, and 8 outcomes supported concave > convex activation [29]. Therefore, more precise methods were needed to delineate the asymmetrical neuromuscular activity in the paraspinal muscles of AIS patients. This study firstly provided evidence of neurogenic abnormalities in the convex side of paraspinal muscles from the level of NMJs structure and molecular expression, but how it affects the activity of neuromuscular function still remains to be explored.

Transformation of muscle fibre types could be induced by many factors, such as development, ageing and nerve injury. A motor neuron and the muscle fibres it supplies comprise a motor unit, and the type of muscle fibres would change when they encounter neurological injury and were controlled by a new motor unit [30]. Higashino et al. found that muscle fibre type conversion occurred in the early stage of both spinal cord transection and peripheral nerve transection in the tibialis anterior muscle of Wistar rats [31]. The phenomenon of muscle fibre type clumping rather than the normal mosaic distribution was also commonly interpreted to reflect nerve injury and muscle reinnervation [32]. Kapchinsky et al. concluded that motor unit remodelling drove the fibre type shift and grouping in the limb muscles of patients with chronic obstructive pulmonary disease [33]. They also found that degeneration of NMJs preceded the changes of muscle fibres in an animal model [33]. In this study, we observed that type I fibre was dominant on the convex side of paraspinal muscles in AIS patients. This result was consistent with previous studies but was always thought to be a secondary outcome of scoliosis [8, 34, 35]. We speculated that it occurred following the neurogenic abnormalities and provided sufficient evidence to support this. Therefore, the potential mechanisms of AIS might be due to subtle neuromuscular dysfunction. It caused the imbalance of bilateral paraspinal muscles, thus driving the onset of scoliosis.

This study has several limitations. First, this study was carried out in a single centre, and the sample size was relatively small. More cases from multi‐centres were needed to validate our results. Second, due to the requirements of medical ethics, we couldn't directly collect samples of nervous tissue from AIS patients during surgery. Therefore, it was hard to figure out the specific causes and mechanisms of neurogenic abnormalities currently. Third, this study didn't analyse the correlation between NMJ's morphology and NCAM's expression. Last, although we elaborated on the morphological disorders of NMJs in the paraspinal muscles, this study failed to explain the functional changes of neuromuscular activities. Previous studies used surface EMG to detect the electrical activities of paraspinal muscles, but their conclusions contradicted each other and could not reach a consensus [29]. Therefore, more advanced methods are needed to accurately measure the neuromuscular function of paraspinal muscles for AIS patients in the future.

## 4 Materials and Methods

### 4.1 Study Participants

The study design was prospective and controlled. AIS patients and age‐matched CS patients who received scoliosis correction surgery from August 2022 to 2023 in our centre were prospectively recruited in this study. Age‐matched teenagers who received posterior spinal surgery due to spinal fracture or lumbar disc herniation were also enrolled as controls. Participants who were reluctant to join the study or declined to provide informed consent were excluded. The whole‐spine MRI was performed to exclude any possible intraspinal abnormality for all patients. The baseline clinical and radiographic information was collected. The magnitude of the scoliosis curve angle was measured by Cobb's method on the full‐spine standing radiographs.

### 4.2 Tissue Sampling

For AIS and CS patients, muscle tissues were sampled from the apical region of bilateral deep paraspinal muscles during posterior scoliosis correction surgery. For non‐scoliosis patients, deep paraspinal muscles were also sampled from a similar vertebral level region. For immunofluorescent staining of NMJs, long‐stripe blocks of tissue (approx. 2 cm in length) were needed because too short muscle fibres were insufficient to detect the location of NMJs on them [36]. After drying excess blood in muscle samples and removing visible fat/connective tissue, muscle samples were fixed overnight in 4% paraformaldehyde (PFA). Then, samples were washed in phosphate‐buffered saline (PBS) three times and stored in PBS until labelling.

### 4.3 Immunofluorescent Staining of NMJs

To label the NMJs, small bundles of 20–30 muscle fibres were first teased from the large whole muscles under a stereomicroscope. NMJs were immunohistochemically labelled according to a standard laboratory protocol for visualising pre‐ and post‐synaptic apparatus [37]. Transfer muscle bundles into 16‐well plates containing 1% bovine serum albumin (BSA) and 0.5% Triton X‐100 and keep them under gentle agitation for 1 h at room temperature (RT). Wash samples 3 times for 5 min with PBS at RT. Incubate samples with the primary antibodies in 5% BSA overnight at 4°C. Wash samples 3 times for 5 min with PBS at RT. Incubate samples with the secondary antibodies in 1% PBS overnight at 4°C. Wash samples 3 times for 5 min with PBS at RT. Place them on a slide with an antifade mounting medium (Aqua‐Poly/Mount) and cover them with a cover slip. Store at −20°C prior to imaging. The primary antibodies were rabbit anti‐neurofilament‐L (1:200, Cell Signaling Technology, #2837S) to label presynaptic axon terminals and rabbit anti‐synapsin‐1 (1:400, Cell Signaling Technology, #5297S) to label synaptic vesicles. The secondary antibodies were Alexa Fluor 488 goat anti‐rabbit Immunoglobulin (1:500, Invitrogen, # A11034) and tetramethylrhodamine‐conjugated α‐bungarotoxin (1:500, Invitrogen, #T1175) to label post‐synaptic acetylcholine receptors (AChRs).

### 4.4 Morphology Analysis of NMJs

Confocal images of NMJs were acquired and analysed using a standardised ‘NMJ‐morph’ workflow [38]. Specifically, a Leica TCS SP8 confocal microscope was used to acquire z‐stack projections of NMJs and their pre‐terminal axon. Images were then analysed on maximum intensity projections of the z‐stacks, using ImageJ software. We measured morphological variables for each NMJ, including pre‐synaptic variables (axon diameter, nerve terminal area, nerve terminal perimeter, number terminal branches, number branch points, total length branches, average length branches, complexity) and post‐synaptic variables (AChR area, AChR perimeter, endplate area, endplate perimeter, endplate diameter, number AChR clusters, average area AChR clusters, fragmentation, compactness, overlap). The exact definition of each variable could be referred to in previous studies [38]. At least 20 NMJs were measured per muscle sample for each patient. The researcher who performed the measurements was blinded to the clinical information.

### 4.5 Location of NMJs

In order to identify the location of the endplate bands, muscle fibres were labelled with α‐bungarotoxin for 5 min [36]. The synapse‐enriched portions of the muscle fibres were then micro‐dissected under a fluorescence microscope. Synapse‐enriched samples, along with synapse‐devoid muscle samples, were then frozen to −80°C prior to gene expression analysis. All muscle labelling and dissection were performed within 1 h post‐sampling to limit the degree of RNA degradation.

### 4.6 Gene Expression Analysis

Total RNA was isolated from muscle samples using TRIzol Reagent (Invitrogen, #15596‐018) according to the manufacturer's instructions and reverse transcribed by MuLV reverse transcriptase (NEB, #M0253L) at 42°C for 60 min. Quantitative PCR reactions were performed with FastStart Universal SYBR Green Master (Roche, #4913914001) in the ABI Q6 real‐time PCR system (ABI). The programme was allowed to function at 95°C for 1 min, followed by 40 cycles of 95°C for 20 s and 60°C for 1 min. GAPDH served as the internal control. The relative fold change was calculated using the 2^−ΔΔCt^ method. The primers of genes of interest for RT‐qPCR are listed in Table S1. The expression of MuSK was also detected by immunofluorescent staining. The primary antibodies were rabbit anti‐MuSK (1:100, Bioss Antibodies, #bs‐6473R), and the secondary antibodies were Alexa Fluor 488 goat anti‐rabbit (1:1000, Invitrogen, # A11034). The mean fluorescence intensity of MuSK was compared between both sides.

### 4.7 Immunofluorescent Staining of Muscle Fibres

Fresh muscle tissues were mounted in frozen section medium (Thermo Fisher Scientific, #6520) and sliced by a cryostat (Leica, #CM1860) to obtain 10 μm cryosections. Then, the sections were mounted on glass slides for further staining. Muscle sections were fixed with 4% PFA and permeabilised with 0.5% Triton X‐100. Following that, 1% BSA was used to block the sections for 1 h at RT. Anti‐laminin (Abcam, #ab11575, 1: 500), anti‐fast myosin heavy chain IIA (Developmental Studies Hybridoma Bank, #SC‐71, 1: 500), anti‐slow myosin heavy chain I (Developmental Studies Hybridoma Bank, #BA‐D5, 1: 100) and anti‐CD56 (Invitrogen, #MA5‐11563, 1:40) were selected as primary antibodies for different staining strategies. Sections were incubated in primary antibodies overnight at 4°C and incubated in appropriate Alexa Fluor 488‐ or 647‐labelled secondary antibodies (Invitrogen, 1:1000) for 1 h at RT. The nuclei were then stained with 4,6‐diamidino‐2‐phenylindole (DAPI), and sections were coverslipped with antifade mounting media (Vector Laboratories, H‐100) for subsequent imaging.

### 4.8 Analysis of Fibre‐Type Grouping

In healthy skeletal muscles, the fast and slow fibres appeared to be randomly arranged, looking like a mosaic pattern [39]. The occurrence of large groups of fibres with the same histochemical properties was referred to as ‘fibre‐type grouping’, and was considered to be evidence of a neuropathological process [30]. To assess the extent of fibre type grouping in a cross section, the ‘enclosed fibre’ was defined as any muscle fibre of a given type surrounded by fibres of the same type only [40]. In each cross‐section, the number of enclosed fibres was counted manually. The prevalence of enclosed fibres (%), reflecting fibre type grouping, was calculated as: 100% × number of enclosed fibres/total number of muscle fibres in a region of interest. The difference in prevalence between bilateral paraspinal muscles was compared.

### 4.9 Statistical Analysis

Data are presented as mean ± standard deviation. Statistical analysis was performed by GraphPad Prism 9 (GraphPad Software Inc., San Diego, CA, USA). The paired *t*‐test was used between concave and convex side of AIS and the one‐way ANOVA was used among three groups. Difference was considered significant with *p* < 0.05.

## 5 Conclusion

This study firstly investigated the morphological and molecular disorders of NMJs in the paraspinal muscles from AIS patients. We found that the neurogenic abnormalities existed in the convex side of the paraspinal muscle, followed by the conversion and grouping of fibre types. This resulted in the imbalance of bilateral paraspinal muscles and might be the potential driver of scoliosis.

## Author Contributions

**Tianyuan Zhang:** formal analysis (lead), methodology (lead), software (equal), validation (equal), writing – original draft (lead), writing – review and editing (lead). **Bin Li:** data curation (equal), formal analysis (equal), investigation (equal), methodology (equal), writing – original draft (equal), writing – review and editing (equal). **Wenyuan Sui:** conceptualization (equal), data curation (equal), formal analysis (equal), methodology (equal), writing – original draft (equal), writing – review and editing (equal). **Xiexiang Shao:** data curation (equal), investigation (equal), methodology (equal), software (equal), validation (equal). **Yaolong Deng:** investigation (equal), resources (equal), supervision (equal), writing – review and editing (equal). **Zifang Zhang:** data curation (equal), resources (equal), validation (equal), visualization (equal). **Jingfan Yang:** data curation (equal), resources (equal), software (equal), supervision (equal). **Zifang Huang:** data curation (equal), resources (equal), supervision (equal), validation (equal). **Sheng Li:** conceptualization (equal), investigation (equal), methodology (equal), validation (equal). **Xin Fu:** conceptualization (equal), investigation (equal), methodology (equal), resources (equal). **Wenjun Yang:** conceptualization (equal), funding acquisition (equal), investigation (equal), methodology (equal), project administration (equal), resources (equal), supervision (equal), writing – review and editing (equal). **Junlin Yang:** conceptualization (equal), funding acquisition (lead), methodology (equal), project administration (lead), resources (equal), supervision (lead), visualization (equal). **Ping Hu:** conceptualization (equal), funding acquisition (equal), methodology (equal), project administration (equal), resources (equal), supervision (equal), writing – review and editing (equal).

## Ethics Statement

This study was approved by the Ethics Committee of Xin Hua Hospital Affiliated to Shanghai Jiao Tong University School of Medicine (No. XHEC‐D‐2019‐093).

## Consent

All cases signed informed consent before their inclusion in the study.

## Conflicts of Interest

The authors declare no conflicts of interest.

## Supporting information


Figure S1.

Table S1.

## Data Availability

The data that support the findings of this study are available from the authors upon reasonable request.

## References

[1] S. L. Weinstein , L. A. Dolan , J. C. Cheng , A. Danielsson , and J. A. Morcuende , “Adolescent Idiopathic Scoliosis,” Lancet 371, no. 9623 (2008): 1527–1537. 18456103 10.1016/S0140-6736(08)60658-3

[2] F. Altaf , A. Gibson , Z. Dannawi , and H. Noordeen , “Adolescent Idiopathic Scoliosis,” BMJ (Clinical Research Edition) 346 (2013): f2508. 10.1136/bmj.f2508 23633006

[3] F. Zaina , J. Wynne , and L. Cohen , “Scoliosis and Spinal Deformities: Twenty Years of Innovations,” European Journal of Physical and Rehabilitation Medicine 59, no. 4 (2023): 502–504. 37746782 10.23736/S1973-9087.23.08218-7 PMC10548475

[4] S. Marya , A. D. Tambe , P. A. Millner , and A. I. Tsirikos , “Adolescent Idiopathic Scoliosis: A Review of Aetiological Theories of a Multifactorial Disease,” Bone & Joint Journal 104‐B, no. 8 (2022): 915–921. 10.1302/0301-620X.104B8.BJJ-2021-1638.R1 35909373

[5] J. C. Cheng , R. M. Castelein , W. C. Chu , et al., “Adolescent Idiopathic Scoliosis,” Nature Reviews. Disease Primers 1 (2015): 15030. 10.1038/nrdp.2015.30 27188385

[6] X. Jiang , F. Liu , M. Zhang , et al., “Advances in Genetic Factors of Adolescent Idiopathic Scoliosis: A Bibliometric Analysis,” Frontiers in Pediatrics 11 (2023): 1301137. 38322243 10.3389/fped.2023.1301137 PMC10845672

[7] W. W. Y. Chan , S. N. Fu , T. F. Chong , et al., “Associations Between Paraspinal Muscle Characteristics and Spinal Curvature in Conservatively Treated Adolescent Idiopathic Scoliosis: A Systematic Review,” Spine Journal 24 (2023): 692–720. 10.1016/j.spinee.2023.11.008 38008187

[8] I. Stetkarova , J. Zamecnik , V. Bocek , P. Vasko , K. Brabec , and M. Krbec , “Electrophysiological and Histological Changes of Paraspinal Muscles in Adolescent Idiopathic Scoliosis,” European Spine Journal 25, no. 10 (2016): 3146–3153. 27246349 10.1007/s00586-016-4628-8

[9] C. Xue , L. Shi , S. C. N. Hui , et al., “Altered White Matter Microstructure in the Corpus Callosum and Its Cerebral Interhemispheric Tracts in Adolescent Idiopathic Scoliosis: Diffusion Tensor Imaging Analysis,” AJNR. American Journal of Neuroradiology 39, no. 6 (2018): 1177–1184. 29674416 10.3174/ajnr.A5634 PMC7410631

[10] L. Shi , D. Wang , S. C. Hui , M. C. Tong , J. C. Cheng , and W. C. Chu , “Volumetric Changes in Cerebellar Regions in Adolescent Idiopathic Scoliosis Compared With Healthy Controls,” Spine Journal 13, no. 12 (2013): 1904–1911. 10.1016/j.spinee.2013.06.045 23988458

[11] D. Wang , L. Shi , W. C. Chu , R. G. Burwell , J. C. Cheng , and A. T. Ahuja , “Abnormal Cerebral Cortical Thinning Pattern in Adolescent Girls With Idiopathic Scoliosis,” NeuroImage 59, no. 2 (2012): 935–942. 21872666 10.1016/j.neuroimage.2011.07.097

[12] Y. Kong , L. Shi , S. C. Hui , et al., “Variation in Anisotropy and Diffusivity Along the Medulla Oblongata and the Whole Spinal Cord in Adolescent Idiopathic Scoliosis: A Pilot Study Using Diffusion Tensor Imaging,” AJNR. American Journal of Neuroradiology 35, no. 8 (2014): 1621–1627. 24788126 10.3174/ajnr.A3912 PMC7964454

[13] J. Domenech , G. García‐Martí , L. Martí‐Bonmatí , C. Barrios , J. M. Tormos , and A. Pascual‐Leone , “Abnormal Activation of the Motor Cortical Network in Idiopathic Scoliosis Demonstrated by Functional MRI,” European Spine Journal 20, no. 7 (2011): 1069–1078. 21499781 10.1007/s00586-011-1776-8 PMC3176702

[14] B. Garg , M. Gupta , N. Mehta , and R. Malhotra , “Influence of Etiology and Onset of Deformity on Spatiotemporal, Kinematic, Kinetic, and Electromyography Gait Variables in Patients With Scoliosis‐A Prospective, Comparative Study,” Spine (Phila PA 1976) 46, no. 6 (2021): 374–382. 33620181 10.1097/BRS.0000000000003796

[15] K. K. L. Lau , K. K. P. Law , K. Y. H. Kwan , J. P. Y. Cheung , K. M. C. Cheung , and A. Y. L. Wong , “Timely Revisit of Proprioceptive Deficits in Adolescent Idiopathic Scoliosis: A Systematic Review and Meta‐Analysis,” Global Spine Journal 12, no. 8 (2022): 1852–1861. 34911378 10.1177/21925682211066824 PMC9609540

[16] Z. Chen , Y. Qiu , W. Ma , B. Qian , and Z. Zhu , “Comparison of Somatosensory Evoked Potentials Between Adolescent Idiopathic Scoliosis and Congenital Scoliosis Without Neural Axis Abnormalities,” Spine Journal 14, no. 7 (2014): 1095–1098. 10.1016/j.spinee.2013.07.465 24099684

[17] W. D. Arnold and B. C. Clark , “Neuromuscular Junction Transmission Failure in Aging and Sarcopenia: The Nexus of the Neurological and Muscular Systems,” Ageing Research Reviews 89 (2023): 101966. 37270145 10.1016/j.arr.2023.101966 PMC10847753

[18] S. Y. Ng and V. Ljubicic , “Recent Insights Into Neuromuscular Junction Biology in Duchenne Muscular Dystrophy: Impacts, Challenges, and Opportunities,” eBioMedicine 61 (2020): 103032. 33039707 10.1016/j.ebiom.2020.103032 PMC7648118

[19] R. I. Wadman , A. F. Vrancken , L. H. van den Berg , and W. L. van der Pol , “Dysfunction of the Neuromuscular Junction in Spinal Muscular Atrophy Types 2 and 3,” Neurology 79, no. 20 (2012): 2050–2055. 23115209 10.1212/WNL.0b013e3182749eca

[20] Z. Feng , S. Lam , E. S. Tenn , et al., “Activation of Muscle‐Specific Kinase (MuSK) Reduces Neuromuscular Defects in the Delta7 Mouse Model of Spinal Muscular Atrophy (SMA),” International Journal of Molecular Sciences 22, no. 15 (2021): 8015. 34360794 10.3390/ijms22158015 PMC8348537

[21] A. Navarro‐Martínez , C. Vicente‐García , and J. J. Carvajal , “NMJ‐Related Diseases Beyond the Congenital Myasthenic Syndromes,” Frontiers in Cell and Development Biology 11 (2023): 1216726. 10.3389/fcell.2023.1216726 PMC10436495 37601107

[22] M. Kinali , S. Messina , E. Mercuri , et al., “Management of Scoliosis in Duchenne Muscular Dystrophy: A Large 10‐Year Retrospective Study,” Developmental Medicine and Child Neurology 48, no. 6 (2006): 513–518. 16700946 10.1017/S0012162206001083

[23] C. A. Wijngaarde , R. C. Brink , F. A. S. de Kort , et al., “Natural Course of Scoliosis and Lifetime Risk of Scoliosis Surgery in Spinal Muscular Atrophy,” Neurology 93, no. 2 (2019): e149–e158. 31164393 10.1212/WNL.0000000000007742

[24] X. Wang , M. Yue , J. P. Y. Cheung , et al., “Impaired Glycine Neurotransmission Causes Adolescent Idiopathic Scoliosis,” Journal of Clinical Investigation 134, no. 2 (2024): e168783. 37962965 10.1172/JCI168783 PMC10786698

[25] S. Aare , S. Spendiff , M. Vuda , et al., “Failed Reinnervation in Aging Skeletal Muscle,” Skeletal Muscle 6, no. 1 (2016): 29. 27588166 10.1186/s13395-016-0101-y PMC5007704

[26] Z. Zhu , Y. Qiu , B. Wang , Y. Yu , B. Qian , and F. Zhu , “Abnormal Spreading and Subunit Expression of Junctional Acetylcholine Receptors of Paraspinal Muscles in Scoliosis Associated With Syringomyelia,” Spine (Phila PA 1976) 32, no. 22 (2007): 2449–2454. 18090084 10.1097/BRS.0b013e3181573d01

[27] M. C. Theroux , R. E. Akins , C. Barone , B. Boyce , F. Miller , and K. W. Dabney , “Neuromuscular Junctions in Cerebral Palsy: Presence of Extrajunctional Acetylcholine Receptors,” Anesthesiology 96, no. 2 (2002): 330–335. 11818764 10.1097/00000542-200202000-00017

[28] Y. Park , J. Y. Ko , J. Y. Jang , S. Lee , J. Beom , and J. S. Ryu , “Asymmetrical Activation and Asymmetrical Weakness as Two Different Mechanisms of Adolescent Idiopathic Scoliosis,” Scientific Reports 11, no. 1 (2021): 17582. 34475442 10.1038/s41598-021-96882-8 PMC8413345

[29] P. T. T. Ng , A. Claus , M. T. Izatt , P. Pivonka , and K. Tucker , “Is Spinal Neuromuscular Function Asymmetrical in Adolescents With Idiopathic Scoliosis Compared to Those Without Scoliosis?: A Narrative Review of Surface EMG Studies,” Journal of Electromyography and Kinesiology 63 (2022): 102640. 35219074 10.1016/j.jelekin.2022.102640

[30] J. Lexell , D. Downham , and M. Sjöström , “Morphological Detection of Neurogenic Muscle Disorders: How Can Statistical Methods Aid Diagnosis?,” Acta Neuropathologica 75, no. 2 (1987): 109–115. 3324621 10.1007/BF00687070

[31] K. Higashino , T. Matsuura , K. Suganuma , K. Yukata , T. Nishisho , and N. Yasui , “Early Changes in Muscle Atrophy and Muscle Fiber Type Conversion After Spinal Cord Transection and Peripheral Nerve Transection in Rats,” Journal of Neuroengineering and Rehabilitation 10 (2013): 46. 23687941 10.1186/1743-0003-10-46 PMC3668998

[32] T. Gordon and J. E. T. de Zepetnek , “Motor Unit and Muscle Fiber Type Grouping After Peripheral Nerve Injury in the Rat,” Experimental Neurology 285 (2016): 24–40. 27594094 10.1016/j.expneurol.2016.08.019

[33] S. Kapchinsky , M. Vuda , K. Miguez , et al., “Smoke‐Induced Neuromuscular Junction Degeneration Precedes the Fibre Type Shift and Atrophy in Chronic Obstructive Pulmonary Disease,” Journal of Physiology 596, no. 14 (2018): 2865–2881. 29663403 10.1113/JP275558 PMC6046075

[34] B. Shahidi , A. Yoo , C. Farnsworth , P. O. Newton , and S. R. Ward , “Paraspinal Muscle Morphology and Composition in Adolescent Idiopathic Scoliosis: A Histological Analysis,” JOR Spine 4, no. 3 (2021): e1169. 34611591 10.1002/jsp2.1169 PMC8479518

[35] X. Shao , J. Chen , J. Yang , et al., “Fiber Type‐Specific Morphological and Cellular Changes of Paraspinal Muscles in Patients With Severe Adolescent Idiopathic Scoliosis,” Medical Science Monitor 26 (2020): e924415. 32778639 10.12659/MSM.924415 PMC7412933

[36] R. A. Jones , C. Harrison , S. L. Eaton , et al., “Cellular and Molecular Anatomy of the Human Neuromuscular Junction,” Cell Reports 21, no. 9 (2017): 2348–2356. 29186674 10.1016/j.celrep.2017.11.008 PMC5723673

[37] I. Boehm , J. Miller , T. M. Wishart , et al., “Neuromuscular Junctions Are Stable in Patients With Cancer Cachexia,” Journal of Clinical Investigation 130, no. 3 (2020): 1461–1465. 31794435 10.1172/JCI128411 PMC7269586

[38] R. A. Jones , C. D. Reich , K. N. Dissanayake , et al., “NMJ‐Morph Reveals Principal Components of Synaptic Morphology Influencing Structure‐Function Relationships at the Neuromuscular Junction,” Open Biology 6, no. 12 (2016): 160240. 27927794 10.1098/rsob.160240 PMC5204123

[39] J. Lexell and D. Y. Downham , “The Occurrence of Fibre‐Type Grouping in Healthy Human Muscle: A Quantitative Study of Cross‐Sections of Whole Vastus Lateralis From Men Between 15 and 83 Years,” Acta Neuropathologica 81, no. 4 (1991): 377–381. 2028741 10.1007/BF00293457

[40] G. A. M. Messa , M. Piasecki , J. Rittweger , et al., “Absence of an Aging‐Related Increase in Fiber Type Grouping in Athletes and Non‐Athletes,” Scandinavian Journal of Medicine & Science in Sports 30, no. 11 (2020): 2057–2069. 32706412 10.1111/sms.13778

