# Supplementary material for: The Neurogenic Abnormities of Paraspinal Muscles Lead to Asymmetry of Fibre Types in Adolescent Idiopathic Scoliosis
Source: J Cell Mol Med. 2025 May 24;29(10):e70619. doi: 10.1111/jcmm.70619 (PMC12102663; doi:10.1111/jcmm.70619)
Supplement: Supplementary file 1 — Figure S1. [file JCMM-29-e70619-s001.docx]

**Supplementary Figure 1**


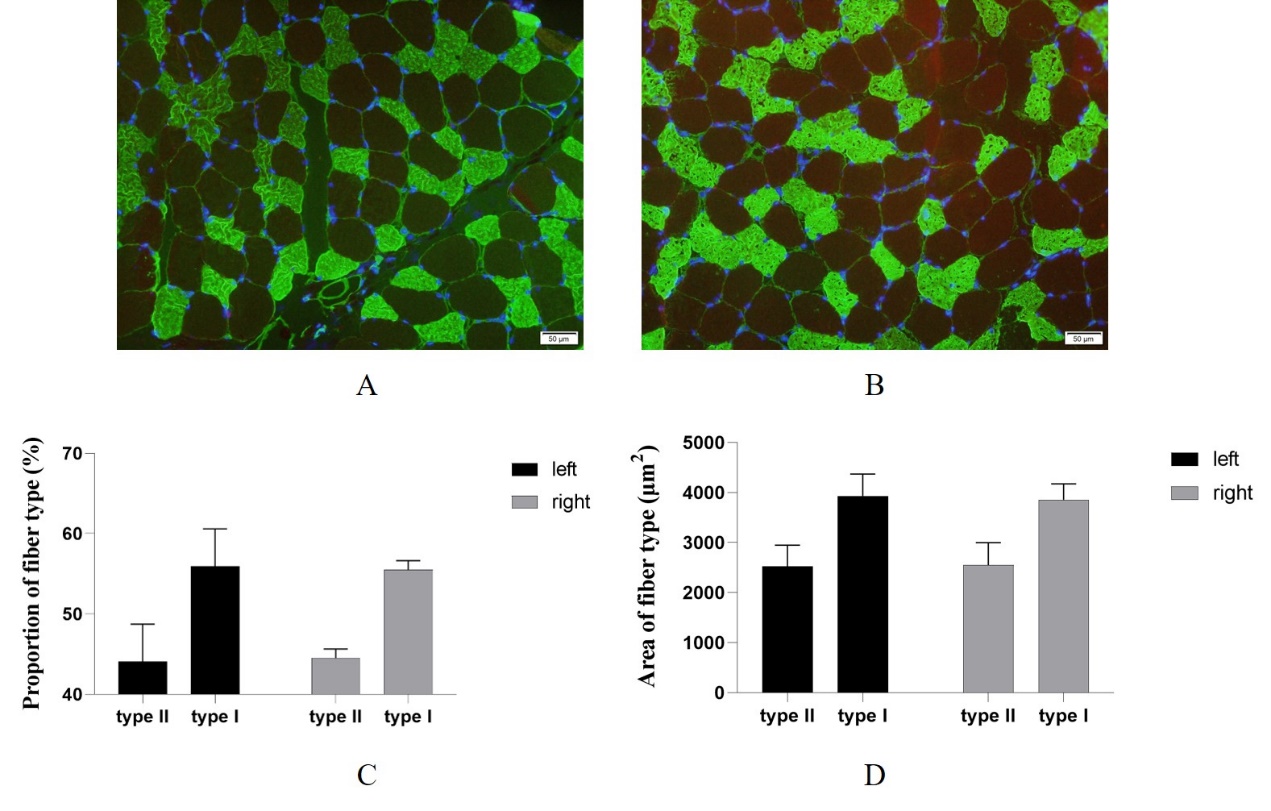


Analysis of proportion and area of fiber types for normal controls. (A) immunofluorescent staining of fiber types in the left side of paraspinal muscles; (B) immunofluorescent staining of fiber types in the right side of paraspinal muscles; (C) comparison of proportion of fiber types between both sides of paraspinal muscles; (D) comparison of area of fiber types between both sides of paraspinal muscles. (green fluorescence stains type II muscle fibers)
