# Supplementary material for: The Neurogenic Abnormities of Paraspinal Muscles Lead to Asymmetry of Fibre Types in Adolescent Idiopathic Scoliosis
Source: J Cell Mol Med. 2025 May 24;29(10):e70619. doi: 10.1111/jcmm.70619 (PMC12102663; doi:10.1111/jcmm.70619)
Supplement: Supplementary file 2 — Table S1. [file JCMM-29-e70619-s002.docx]

**Supplementary Table 1**

| Gene | Primer |
| --- | --- |
| Human GAPDH-F | 5'-AGGTCGGAGTCAACGGATTT-3' |
| Human GAPDH-R | 5'-GACGGTGCCATGGAATTTGC-3' |
| Human MYH1-F | 5'-TCCAAAGCCAAGGGAAACCT-3' |
| Human MYH1-R | 5'-CCCCTCGAGAGCTGTGAAAC-3' |
| Human MYH2-F | 5'-TCTCCAAAGCCAAGGGAAACC-3' |
| Human MYH2-R | 5'-AACTGAGACACCAGAGCTTCC-3' |
| Human MYH4-F | 5'-TCTCCAAAGCCAAGGCAAAC-3' |
| Human MYH4-R | 5'-CCTTCTGGGCTGACAACTCAT-3' |
| Human MYH7-F | 5'-GGGAGATTCGGAGATGGCAG-3' |
| Human MYH7-R | 5'-TTCACGGTCACTGTCTTGCC-3' |
| Human TNNI1-F | 5'-GAAGACACAGAGAAGGAGCGG-3' |
| Human TNNI1-R | 5'-ACTTGGCGGCATCAAACATC-3' |
| Human TNNC1-F | 5'-ACATCTACAAGGCTGCGGTAG-3' |
| Human TNNC1-R | 5'-CGGAAGAGGTCAGACAGCTC-3' |
| Human TNNT1-F | 5'-TACCTGGTCAAGGCAGAACA-3' |
| Human TNNT1-R | 5'-TGTGATGGAGGCAGCCAG-3' |
| Human TNNI2-F | 5'-GCAGCACCTGAAGAGTGTGAT-3' |
| Human TNNI2-R | 5'-TTCAGCCGCATCGATCTTGG-3' |
| Human TNNC2-F | 5'-GATGGTGGTGGGGACATCAG-3' |
| Human TNNC2-R | 5'-TAGCCGTCTGCATTCCTGTC-3' |
| Human TNNT3-F | 5'-AGAGGCAAGAAGCAGACAGC-3' |
| Human TNNT3-R | 5'-TTGGCCTTGTCCCTCAGTTT-3' |
| Human Ncam1-F | 5'-AAGGGCAGATGGGAGAGGAT-3' |
| Human Ncam1-R | 5'-TGCCGTTGGCTGGGATG-3' |
| Human MuSK-F | 5'-TTTGCTGTCCGTGCCAGAATGC-3' |
| Human MuSK-R | 5'-GGCTTTGAGGACGTCATTGGTG-3' |
| Human Lrp4-F | 5'-GTGTGGCAGAACCTTGACAGTC-3' |
| Human Lrp4-R | 5'-ACCGCTCTAACTTGGCATTCTCC-3' |
| Human Rapsyn-F | 5'-CGCTGGACAAGGCTCTGG-3' |
| Human Rapsyn-R | 5'-AGTAGAGCTCCGTCTCCTCC-3' |
| Human Dok7-F | 5'-GGACGGCAAGAAGTGGAAG-3' |
| Human Dok7-R | 5'-GATGCAGTCGAACAGGAAGC-3' |
| Human Chrng-F | 5'-GCCGGATATCGTGCTGGAG-3' |
| Human Chrng-R | 5'-GCCCACTCCCCATTCTCTGT-3' |
| Human Chrna1-F | 5'-TCCTGCTCTTTAGCCTTTGCTC-3' |
| Human Chrna1-R | 5'-AGATCTACCATGTCACCCTGTTT-3' |
| Human Chrne-F | 5'-CTTGGGGTCCTGCTCCTCTTG-3' |
| Human Chrne-R | 5'-TCCTTGCTGTAGTTGAGTCGG-3' |

**Supplementary Table 2**

|  | AIS Concave | AIS Convex | P Value (paired t-test,) | CS Concave | CS Convex | P Value (paired t-test,) | Normal Controls | P Value (one-way ANOVA) |
| --- | --- | --- | --- | --- | --- | --- | --- | --- |
| Pre-synaptic |  |  |  |  |  |  |  |  |
| Axon Diameter (μm) | 1.67±0.41 | 1.62±0.35 | 0.732 | 1.92±0.16 | 1.79±0.17 | 0.371 | 1.79±0.22 | 0.295 |
| Nerve Terminal Perimeter (μm) | 75.51±16.78 | 65.42±16.47 | 0.007* | 82.25±11.71 | 76.59±13.56 | 0.597 | 88.45±14.41 | 0.057 |
| Nerve Terminal Area (μm^2^) | 93.72±27.35 | 68.23±13.19 | 0.023* | 103.94±30.37 | 94.34±27.07 | 0.547 | 114.42±29.18 | 0.011* |
| Number of Terminal Branches | 6.03±2.13 | 6.25±2.21 | 0.713 | 6.25±2.04 | 7.33±0.81 | 0.432 | 8.13±1.44 | 0.241 |
| Number of Branch Points | 2.32±0.55 | 1.59±0.38 | 0.014* | 2.18±0.24 | 2.65±0.64 | 0.225 | 3.27±0.40 | 0.002* |
| Total Length of Branches (μm) | 27.88±6.73 | 23.24±3.07 | 0.093 | 29.47±5.13 | 30.19±4.94 | 0.860 | 36.59±7.57 | 0.019* |
| Average Length of Branches (μm) | 5.35±1.14 | 4.73±1.17 | 0.212 | 5.76±1.52 | 5.40±1.55 | 0.770 | 5.31±1.69 | 0.858 |
| Complexity | 2.37±0.45 | 2.14±0.3 | 0.147 | 2.21±0.28 | 2.47±0.20 | 0.245 | 2.78±0.35 | 0.048* |
| Post-synaptic |  |  |  |  |  |  |  |  |
| AChR Perimeter (μm) | 99.10±39.05 | 103.30±40.59 | 0.613 | 112.82±33.23 | 105.81±35.09 | 0.802 | 102.69±34.35 | 0.851 |
| AChR Area (μm^2^) | 123.64±29.31 | 153.64±27.54 | 0.007* | 145.32±30.74 | 124.52±17.70 | 0.333 | 148.67±20.47 | 0.119 |
| Endplate Diameter (μm) | 24.17±3.92 | 25.07±2.13 | 0.461 | 28.06±4.57 | 27.03±4.90 | 0.797 | 25.10±6.57 | 0.492 |
| Endplate Perimeter (μm) | 69.55±5.00 | 70.76±7.70 | 0.628 | 76.02±6.91 | 72.01±7.60 | 0.451 | 69.67±18.42 | 0.604 |
| Endplate Area (μm^2^) | 241.45±65.36 | 274.21±46.89 | 0.156 | 292.48±47.31 | 259.02±53.31 | 0.272 | 290.18±66.22 | 0.327 |
| Compactness (%) | 52.86±6.64 | 56.24±8.74 | 0.170 | 50.62±7.77 | 54.41±6.26 | 0.388 | 52.36±6.69 | 0.621 |
| Number of AChR Clusters | 3.22±1.01 | 2.84±0.79 | 0.180 | 2.93±0.56 | 3.09±0.63 | 0.712 | 3.48±0.42 | 0.461 |
| Average Area of AChR Clusters (μm^2^) | 56.14±17.26 | 71.89±18.24 | 0.029* | 66.20±17.84 | 54.69±13.43 | 0.275 | 53.69±17.44 | 0.143 |
| Fragmentation | 0.53±0.15 | 0.49±0.12 | 0.309 | 0.56±0.10 | 0.56±0.14 | 0.999 | 0.59±0.06 | 0.451 |
| Integrated |  |  |  |  |  |  |  |  |
| Overlap (%) | 50.32±7.64 | 33.85±8.40 | <0.001* | 43.50±7.88 | 42.56±13.39 | 0.827 | 53.45±7.84 | 0.007* |

(AIS=adolescent idiopathic scoliosis, CS=congenital scoliosis, *means significantly different)

**Supplementary Table 3**

|  | Left | Right | P value |
| --- | --- | --- | --- |
| Proportion of type I fiber (%) | 55.9±3.8 | 55.5±0.9 | 0.858 |
| Proportion of type II fiber (%) | 44.1±3.8 | 44.5±0.9 | 0.858 |
| P value | 0.036 | <0.001 |  |
| Area of type I fiber (μm2) | 3932.5±357.3 | 3858.1±258.6 | 0.469 |
| Area of type II fiber (μm2) | 2527.0±344.4 | 2546.1±368.8 | 0.627 |
| P value | 0.016 | 0.018 |  |
